# Quantitative Structure–Activity Relationships of Natural-Product-Inspired, Aminoalkyl-Substituted 1-Benzopyrans as Novel Antiplasmodial Agents

**DOI:** 10.3390/molecules26175249

**Published:** 2021-08-30

**Authors:** Friederike M. Wunsch, Bernhard Wünsch, Freddy A. Bernal, Thomas J. Schmidt

**Affiliations:** 1 Institute of Pharmaceutical Biology and Phytochemistry (IPBP), University of Münster, PharmaCampus, Corrensstrasse 48, 48149 Münster, Germany; FriederikeWunsch@gmx.de (F.M.W.); Freddy.Bernal@hki-jena.de (F.A.B.); 2 Institute of Pharmaceutical and Medicinal Chemistry (IPMC), University of Münster, PharmaCampus, Corrensstrasse 48, 48149 Münster, Germany; wuensch@uni-muenster.de; 3 Transfer Group Anti-Infectives, Leibniz Institute for Natural Product Research and Infection Biology—Hans Knöll Institute (HKI), Beutenbergstraße 11a, 07745 Jena, Germany

**Keywords:** aminoalkyl benzopyran, aminoalkyl chromene, aminoalkyl chromane, *Plasmodium falciparum*, malaria, structure–activity relationship, 3D-QSAR

## Abstract

On the basis of the finding that various aminoalkyl-substituted chromene and chromane derivatives possess strong and highly selective in vitro bioactivity against *Plasmodium falciparum*, the pathogen responsible for tropical malaria, we performed a structure–activity relationship study for such compounds. With structures and activity data of 52 congeneric compounds from our recent studies, we performed a three-dimensional quantitative structure–activity relationship (3D-QSAR) study using the comparative molecular field analysis (CoMFA) approach as implemented in the Open3DQSAR software. The resulting model displayed excellent internal and good external predictive power as well as good robustness. Besides insights into the molecular interactions and structural features influencing the antiplasmodial activity, this model now provides the possibility to predict the activity of further untested compounds to guide our further synthetic efforts to develop even more potent antiplasmodial chromenes/chromanes.

## 1. Introduction

Malaria is one of the most life-threatening infectious diseases. It is caused by eukaryotic blood parasites of the genus *Plasmodium* and spread by an insect vector, female *Anopheles* mosquito. The World Health Organization (WHO) estimated a worldwide 229 million cases and 409,000 deaths by malaria in 2019 [1]. The decline in these figures observed in the last decade has been slowed down, at least partly because of the COVID-19 pandemic, so that higher than expected malaria morbidity and mortality have been predicted for coming years by the WHO [1].

Increasing resistance of the parasites, in particular *Plasmodium falciparum* (*Pf*) causing tropical malaria, against existing therapies leads to a rise in treatment failures. Continuous efforts are therefore necessary to discover new chemical entities with antiplasmodial activity.

2*H*-Chromene (2*H*-1-benzopyran) and chromane (3,4-dihydro-2*H*-1-benzopyran) derivatives with aminoalkyl substituents at C-6 were recently discovered by some of us to possess strong and selective activity against *Pf* [2,3,4]. Starting from the natural product encecalol angelate (compound **51** in the present study, see Table 1), which had attracted our attention in a natural product-oriented search for chemical entities with antiprotozoal activity [5,6], a series of over 50 congeneric compounds of this type was synthesized and tested for antiprotozoal activity up to the present. Several of these compounds have demonstrated impressive in vitro antiplasmodial activity at half-maximal inhibitory concentration (IC_50_) values in the nanomolar range, accompanied by high selective toxicity towards the parasites in comparison with mammalian cells [2,3,4].

With the structures and activity data of 52 congeneric compounds of this series in hands, we then performed a three-dimensional quantitative structure–activity relationship (3D-QSAR) study. For this purpose, we chose an approach based on comparative molecular field analysis (CoMFA), in which the compounds’ interaction energies with virtual probes in the surrounding space were calculated as molecular interaction fields (MIFs), which were then analyzed by partial least squares (PLS) regression modeling for correlations with the activity data. Here, we present the 3D-QSAR model resulting from this study.

## 2. Results and Discussion

### 2.1. 3D-QSAR Modeling

**Figure 1:**
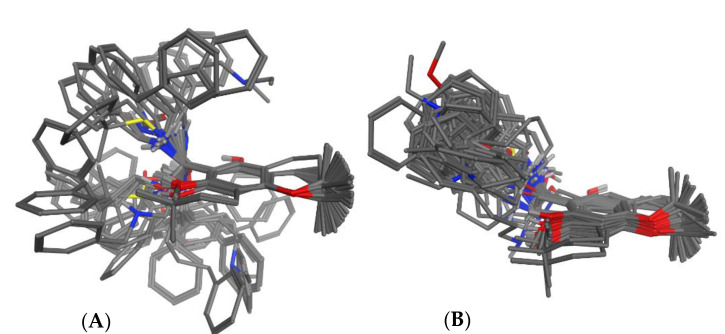
Molecular alignments generated before 3D-QSAR modeling: (**A**): manual alignment of lowest-energy conformers, based on superposition of the benzopyran core; (**B**): alignment based on pharmacophore properties generated with the Pharmacophore Elucidate function of MOE. The latter was used for the 3D-QSAR models.

Three-dimensional (3D) molecular models of 52 congeneric aminoalkyl chromenes and chromanes from our previous studies ([2,3,4,6], for structures, see Table 1) were created and geometry optimized with the MMFF94x force field as implemented in the Molecular Operations Environment (MOE, The Chemical Computing Group, Montreal, QC, Canada). For each structure containing a basic amino group, we created a model of the unprotonated base as well as for the protonated form. The neutral bases and the protonated salts were treated as separate data sets. Since 3D-QSAR and the CoMFA method is known to be very sensitive to molecular alignment, various structural alignments were thus performed (see the Material and Methods section). An automatic alignment based on pharmacophore properties as implemented in MOE, on visual inspection, turned out to yield the most coherent overlap between the structures (see Figure 1B). A manual superposition based on five atoms within the benzofuran core (Figure 1A) as well as an automatic alignment using the Open3DAlign program (not shown) yielded much less coherent results. The aligned molecular models were exported to the Open3DQSAR software [7]. The biological activity data (originally determined in vitro as half-maximal inhibitory concentrations (IC_50_ values against *Pf* in µmol/L) were transformed to pIC_50_ values, i.e., the negative decadic logarithm of the IC_50_ in mol/L, and combined with the structures for the 3D-QSAR study. In the 3D-QSAR analyses, molecular interaction fields (MIFs) were generated with an electrostatic and a steric probe (for details, see the Materials and Methods section) and the MIF data analyzed for correlations with activity using partial least squares (PLS) regression and the variable selection methods implemented in Open3DQSAR. Each 3D-QSAR model was validated with respect to its internal predictivity by leave-one-out cross validation (LOO-CV) and for its external predictivity by activity predictions for the test set compounds. A series of models using all 52 structures as training set was initially computed in order to assess (a) the best molecular alignment and (b) the influence of protonation on the model quality. It was found that the pharmacophore-based alignment of the non-protonated structures (Figure 1B) obtained with MOE yielded the best model. It should be noted that the data set with protonated structures yielded models of much inferior statistical quality so that only the non-protonated structures were further considered. In order to assess the 3D-QSAR models’ external predictivity, we divided the compounds into 20 different training (n = 39) and test sets (n = 14) using different splitting schemes (A-T, see the Supplementary Materials). Considering various such splits of the data sets is also useful in terms of assessing the stability of the model, i.e., its sensitivity to the composition of the training set. All models obtained with the various training/test sets for the non-protonated molecules in the pharmacophore-based alignment were compared with respect to their internal and external predictivity (coefficients of determination for the internal predictions during LOO-CV (*Q*^2^) as well as for test set predictions (*P*^2^)). The results are shown in Table 2.

All 20 subset divisions yielded models with high *R*^2^ and high or at least acceptable *Q*^2^ values. The average number of significant PLS components (PC) was 4.8, i.e., most models comprised five significant components. The average values of *R*^2^ (coefficient of determination for the training set calibration data), *Q*^2^, and *P*^2^ among the 20 models were 0.98, 0.79, and 0.59, respectively. Low relative standard deviations (<10%) of these values indicate low sensitivity to the setup of the training and test sets.

### 2.2. Assessment of Model Robustness and Applicability Domain (AD)

In order to apply more rigorous validation criteria beyond cross validation, we applied the progressive scrambling method as implemented in Open3DQSAR, originally proposed by Clark and Fox [8], to models yielding both *Q*^2^ > 0.75 and *P*^2^ > 0.65 (seven models, highlighted in bold letters in Table 1). This method yields a *Q*_0_^2^* value that is a measure for model robustness and can be interpreted in analogy to the normal *Q*^2^ (i.e., the closer to 1, the better) [8]. The average *Q*_0_^2^* of the seven models investigated in this way was 0.73, with model K yielding the best value of 0.77. Model K was hence considered the best model obtained in this study. A plot of predicted vs. measured activity data in model K is shown in Figure S1.

**Figure 2:**
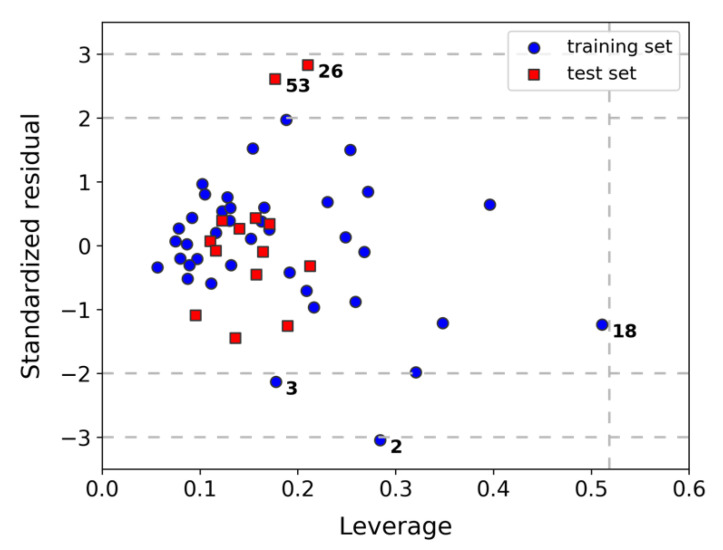
Williams plot for model K. Horizontal dashed lines represent 2σ and 3σ for the standardized residuals. Vertical dashed line represents h*.

Following the recommendations of the OECD (Organization for Economic Cooperation and Development) [9], definition of the AD for model K was achieved through the leverage method (a distance-based method) [10,11,12,13]. The corresponding Williams plot is shown in Figure 2. It can be observed that none of the compounds exceeded the critical leverage, h*, whereupon all the compounds can be characterized as being within the AD of the model. However, compound **18** appeared at a relatively high leverage value, which would indicate that it has a strong influence on the model (being part of the training set). On the other hand, two compounds from the training set (**2** and **3**) and two from the test set (**26** and **53**) showed high error in their predictions (residuals above 2σ). For the former group, the activity was underestimated, whereas for the latter group, it was overestimated. The limited capacity of the model to correctly predict their antiplasmodial activity might be simply related to the fact that **2**, **3**, and **53** possess extreme activity values. In the case of **26**, the excessively overestimated prediction may suggest that this compound behaves somewhat differently compared to the rest, becoming therefore an outlier to the model.

The model’s statistical quality in terms of predictive capability, robustness, and applicability can thus be considered sufficient to make reasonable activity predictions for untested compounds.

### 2.3. Interpretation of 3D-QSAR Model K

**Figure 3:**
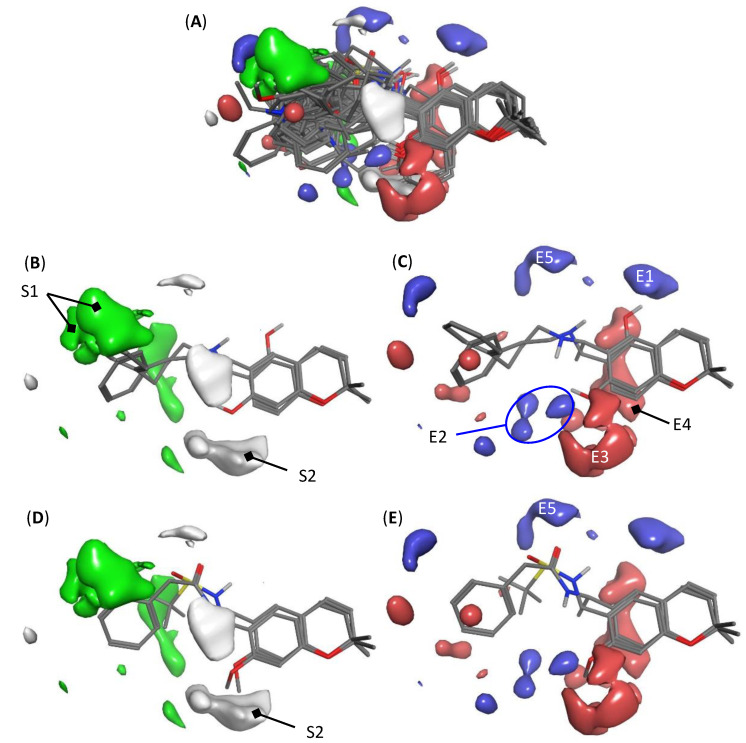
Comparison of MIF regions of the 3D-QSAR model K with strong impact on antiplasmodial activity. In the structures, only polar hydrogens are shown. Steric interactions are shown in green and white, indicating positive and negative impact of steric bulk/Van der Waals interactions on activity, respectively. Electrostatic interactions are shown in blue and red. Blue indicates regions where interaction of positive partial charge (electron deficient partial structure) on the ligand has a positive impact, red indicates regions where interaction of a negative partial charge (electron rich structure element) on the ligand has a positive impact on activity, and vice versa for detrimental effects. (**A**) Superposition of all compounds; (**B**,**C**) superposition of the most active congeners, **1** and **2** (pIC_50_ = 8.0 in both cases); (**D**,**E**) compounds with very low activity, **49** and **52** (pIC_50_ = 4.9 and 4.5, respectively).

3D-QSAR based on comparative molecular field analysis has the advantage that the molecular interactions contributing significantly to the investigated biological activity can easily be visualized by mapping the PLS coefficients of the most relevant regions in the MIFs back into the space surrounding the molecules. Thus, visual inspection of the compounds’ structural features that favorably or unfavorably contribute the activity is rather straightforward. Such MIF-coefficient plots obtained from model K are depicted in Figure 3A for the total molecular ensemble as well as for the two most active congeners and two compounds with very low activity in the data set (compounds **1** and **2**, Figure 3B,C, and compounds **49** and **53**, Figure 3D,E).

It should be mentioned here that variables from the steric and electrostatic MIFs contributed 65 and 35% to the overall model, respectively. Thus, it can be expected that steric interactions are more important for the interference of these compounds with a putative common target structure in plasmodia.

The isolated steric maps (Figure 3B,D) showed a rather large green region (labelled S1 in the figure) in which steric/hydrophobic interactions, resulting mainly from the aromatic moiety in the side chain, led to an enhancement of activity. Compounds not possessing an aromatic system in this region generally had weak activity. On the other hand, a relatively large white contour, besides two smaller ones, indicated that steric bulk in the region denoted S2 will decrease activity. In the steric interactions plot showing compounds **49** and **52**, very weak antiplasmodial agents (Figure 3D), e.g., the methoxy substituent in position R^2^ (compare Table 1), interacted with region S2 so that this may in part be held responsible for the low activity. Similarly, compounds of skeletal type C in Table 1 with an additional pyran ring extended this ring into region S2. All these compounds were only of medium activity (pIC_50_ < 7).

In the electrostatic interaction maps, favorable interactions with the blue regions E1 and E2 were noticed with the OH protons at positions R^2^ and R^3^ of the benzopyran core (compare Table 1). This is, of course, in line with the initial observation of Harel et al. [2,3], in that free phenols of this type had much stronger antiplasmodial activity than the methoxy-substituted congeners. This is evident in Figure 3B, where the structures of the two most active phenols (compound **1** with R^2^ = OH and compound **2** with R^3^ = OH) are shown with their OH protons pointing in the directions of E2 and E1, respectively. Two relatively large red contours (E3 and E4) on both sides of the benzene ring of the core structure indicate activity-enhancing electrostatic interactions with the oxygen atoms of the phenolic OH groups and, possibly, with the mentioned aromatic system, which is more electron-rich in case of free phenols than in case of methoxylated chromanes/chromenes. A further region of interest is E5, which is responsible for detrimental effects of electron rich groups, such as, e.g., the carbonyl and sulfinyl oxygen atoms in case of the two depicted compounds (sulfinamide **49** and carboxamide **52**, respectively). Amino-substituted compounds of this series were, consistently, generally found to be much more active than ester, ether, or amide derivatives [2]. Some smaller regions of electrostatic interactions existed around the side chain aromatic system, which were rather difficult to attribute with general SAR features and rather reflected peculiarities of single compounds of the series.

Overall, the main features of the interaction maps of model K (which were found to be very similar to those of the other six “good” models (C, E, G, J, M, and T in Table 2)) were in very good agreement with the general qualitative SARs established earlier. Naturally, however, this 3D-QSAR model lent itself to a quantitative meaning to each of these structural contributions to activity and provided, as shown above, a means of predicting the activity of untested congeners with good certainty. The model can thus guide further synthetic efforts in a rational manner.

## 3. Materials and Methods

### 3.1. 3D Molecular Models

Molecular models of all compounds were generated with MOE v. 2018.01 (Chemical Computing Group, Montreal, QC, Canada). The biological activity of most compounds with a center of chirality at C-1′ in the side chain had been determined with racemic mixtures. In several cases where enantiomerically pure compounds had been obtained and tested, the C-1′-*R*-enantiomer was found to be much more active (e.g., compounds (*R*)-**4** and (*S*)-**26** (ΔpIC_50_ = 1.54) and to a lesser extent (*R*)-**19** and (*S*)-**24** (ΔpIC_50_ = 0.42)), so that the other compounds were built in the form of their *R*-enantiomers. In the case of one compound (**12k** in [2]), where an additional center of chirality was present in the side chain, structures **36** and **37** were considered separately: both *R*-configured at C-1′ but *R*- and *S*- configured, respectively, at C-3′, using the same pIC_50_ value for both structures.

The models were geometry optimized with the MMFF94x force field implemented in MOE.

### 3.2. Superposition of Molecular Models

#### 3.2.1. Manual Superposition 

The molecules were individually aligned by superposing five atoms of the chromane/chromene skeleton (O-1, C-4, C-4a, C-6, C-8) of the most active compound **1**. The resulting superposition is shown in Figure 1A. It showed very divergent orientation of the side chains, and therefore it was not considered a good starting point for 3D-QSAR modeling.

#### 3.2.2. Automatic Superposition Based on Pharmacophoric Properties 

The module “pharmacophore elucidate” of MOE was used with default settings. Compound **1** was used as template. This calculation in MOE yielded a series of possible alignments scored by coverage of automatically generated 3-, 4-, and 5-point pharmacophores. Visual inspection of these alignments focusing on a coherent positioning of the side chains led us to select the superposition shown in Figure 1B for the 3D-QSAR modeling. The compounds were divided into 20 different training sets and test sets with 39 (74%) and 14 (26%) compounds, respectively, using various schemes. The assignment of compounds to the individual training and test sets is reported in Table S1.

### 3.3. 3D-QSAR Modeling

The aligned molecular models were exported from MOE in SD format, including the bioactivity data (pIC_50_ = −log IC_50_ (mol/L) and read into Open3DQSAR (v. 2.3; P. Tosco and T. Balle, http://open3dqsar.sourceforge.net/ accessed on 28 August 2021; [7]). The box size around the molecular ensemble (23 × 26 × 18 Å) and the grid step size (1.0 Å) were chosen as suggested by the software. Molecular interaction fields were then calculated with a steric probe (an sp^3^ alkyl carbon) as well as an electrostatic probe (a sizeless positive point of charge +1). The number of variables was reduced by applying *cutoff* limits (±30 kcal/mol) and elimination of zero value variables. In order to assign equal weights for the steric and electrostatic interaction fields, which naturally comprise variables with rather different energy values, we submitted the remaining variables to the block unscaled weighting algorithm implemented in Open3DQSAR. Then, a variable selection procedure using Smart Region Definition and Fractional Factorial Design, both as implemented in the Open3DQSAR software, was carried out (thus, e.g., of 28,512 calculated MIF variables, 1737 were used in model K). 3D-QSAR models were then computed with the various training sets by partial least squares (PLS) regression. The best model K (see below) consisted of 797 steric and 940 electrostatic variables, which contributed 65 and 35%, respectively, to the overall model.

### 3.4. Model Validation

Each PLS model was internally cross-validated by LOO-CV. The resulting coefficients of determination for the predicted vs. experimental pIC_50_ values (*R*^2^ for the model calibration, *Q*^2^ for predictions in LOO-CV) along with the standardized errors of calibration, SDEC, and internal prediction, SDEP_int_, are reported in Table 2. External validation was performed in each case by calculating predicted values for the respective test sets. Coefficients of determination for the predicted vs. experimental pIC_50_ values of the test set (*P*^2^) and standardized errors of external predictions (SDEP_ext_) are reported in Table 2. All of the 20 different training/test set divisions provided statistically reasonable models (Table 2). Those yielding both *Q*^2^ > 0.75 and *P*^2^ > 0.65 were further investigated by the progressive scrambling method [8] as implemented in Open3DQSAR. A critical value of s = 0.85 was used, and a polynomial of order 2 was used for curve fitting [8]. The “fitted q^2^ values” as output by the software (Q_s_^2^* in [8]) were normalized by dividing by s to yield the *Q*_0_^2^* values as recommended in [8]. These values are also reported in Table 2. On the basis of its *Q*_0_^2^* value, model K was somewhat superior to the other models and hence chosen as the “best model”.

### 3.5. Assessment of the Applicability Domain (AD) of Model K

AD definition was carried out by the leverage method [10,11,12,13]. Leverages retrieved from the PLS regression of model K and standardized residuals for the predicted antiplasmodial activity were used to build the corresponding Williams plot. The critical leverage h* was used to evaluate whether a compound could be included or not within AD (h* = 3*w*/*N*, where *w* is the total sum of leverages and *N* is the number of compounds). 

### 3.6. Model Visualization

The PLS coefficients of model K were exported to MOE and visualized as MIF contours using the module “grid analyzer”. The graphic representations shown in Figure 3 were generated with isocontour values at PLS coefficients of −0.0009 (white) and +0.0009 (green) for the steric field and −0.00058 (red) and +0.00058 (blue) for the electrostatic field.

## 4. Conclusions

The 3D-QSAR study presented here resulted in a statistically sound model with good predictive capability and explanatory value. The various structural features with major influence on antiplasmodial activity can thus be further explored by in silico design of hitherto untested congeners. The model can then be used to predict the activity of such new chemical entities in order to guide further synthetic efforts. Studies in this direction are under way.

## Figures and Tables

**Table 1:**
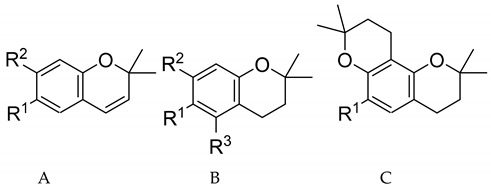
Structures and activity against *Plasmodium falciparum* (pIC_50_) values of the compounds under study. Note that the compounds are tabulated and numbered in the order of decreasing activity. Unless otherwise stated, the C-1′ (*R*)-enantiomer was used in case of compounds with a chiral center at this position (see the Materials and Methods section).

Compound Number	Number in [Reference]	Type	R^1^	R^2^	R^3^	pIC_50_
1	**15b** [2]	A	Ph(CH_2_)_3_NHCH(CH_3_)‒	OH	-	8.00
2	**8b** [4]	B	Ph(CH_2_)_2_NHCH_2_‒	-	OH	8.00
3	**19c** [4]	B	Ph(CH_2_)_3_NHCH_2_CH(OH)‒	OH	H	7.77
4	*R*-**2** [3]	A	PhCH_2_NHC*H(CH_3_)‒ [*(*R*)]	OH	-	7.59
5	**9c** [4]	B	Ph(CH_2_)_3_NHCH_2_‒	OH	H	7.42
6	**8a** [4]	B	PhCH_2_NHCH_2_‒	-	OH	7.28
7	**9d** [4]	B	Ph(CH_2_)_4_NHCH_2_‒	OH	H	7.15
8	**10b** [4]	A	Ph(CH_2_)_2_NHCH_2_‒	OH	H	7.12
9	**10d** [4]	A	Ph(CH_2_)_4_NHCH_2_‒	OH	H	7.10
10	**10c** [4]	A	Ph(CH_2_)_3_NHCH_2_‒	OH	H	7.09
11	**9b** [4]	B	Ph(CH_2_)_2_NHCH_2_‒	OH	H	7.09
12	**9a** [4]	B	PhCH_2_NHCH_2_‒	OH	H	7.00
13	**19a** [4]	B	PhCH_2_NHCH_2_CH(OH)‒	OH	H	7.00
14	**8c** [4]	B	Ph(CH_2_)_3_NHCH_2_‒	H	OH	6.82
15	**10a** [4]	A	PhCH_2_NHCH_2_‒	OH	H	6.80
16	**8d** [4]	B	Ph(CH_2_)_4_NHCH_2_‒	H	OH	6.75
17	**12d** [4]	C	PhCH_2_NHCH_2_‒	-	-	6.68
18	**12b** [4]	C	Ph(CH_2_)_2_NHCH_2_‒	-	-	6.62
19	*R*-**1** [3]	A	PhCH_2_NHC*H(CH_3_)‒ [*(*R*)]	OCH_3_	-	6.55
20	**12c** [4]	C	Ph(CH_2_)_3_NHCH_2_‒	-	-	6.43
21	**12g** [2]	A	PhC*H(CH_3_)NHCH(CH_3_)‒ [*(*S*)]	OCH_3_	-	6.43
22	**12a** [4]	C	PhCH_2_NHCH_2_‒	-	-	6.33
23	**12f** [2]	A	PhC*H(CH_3_)NHCH(CH_3_)‒ [*(*R*)]	OCH_3_	-	6.14
24	*S*-**1** [3]	A	PhCH_2_NHC*H(CH_3_)‒ [*(S)]	OCH_3_	-	6.13
25	**12c** [2]	A	PhCH_2_NHCH(CH_3_)‒	OCH_3_	-	6.07
26	*S*-**2** [3]	A	PhCH_2_NHC*H(CH_3_)‒ [*(S)]	OH	-	6.05
27	**12e** [2]	A	*p*-OCH_3_-PhCH_2_NHCH(CH_3_)‒	OCH_3_	-	6.03
28	**25** [4]	B	PhCH_2_NH(CH_2_)_3_‒	OH	H	6.01
29	**12o** [2]	A	Ph(CH_2_)_3_N(CH)_3_CH(CH_3_)‒	OCH_3_	-	5.91
30	*R*-**10** [3]	A	PhCH_2_N(CH_3_)C*H(CH_3_)‒ [*(*R*)]	OCH_3_	-	5.91
31	*S*-**10** [3]	A	PhCH_2_N(CH_3_)C*H(CH_3_)‒ [*(*S*)]	OCH_3_	-	5.89
32	**14d** [2]	B	Ph(CH_2_)_4_NHCH(CH_3_)‒	OCH_3_	H	5.87
33	**12d** [2]	A	Ph(CH_2_)_4_NHCH(CH_3_)‒	OCH_3_	-	5.85
34	**14a** [2]	B	PhCH_2_NHCH(CH_3_)‒	OCH_3_	H	5.82
35	**14c** [2]	B	Ph(CH_2_)_3_NHCH(CH_3_)‒	OCH_3_	H	5.67
36	**12k** [2] ^a^	A	(C_2_H_5_)_2_N(CH_2_)_3_C*(CH_3_)NHC**H(CH_3_)‒ [*(*S*), **(*R*)]	OCH_3_	-	5.62
37	**12k** [2] ^a^	A	(C_2_H_5_)_2_N(CH_2_)_3_C*(CH_3_)NHC**H(CH_3_)‒ [*(*R*), **(*R*)]	OCH_3_	-	5.62
38	**12b** [2]	A	Ph(CH_2_)_2_NHCH(CH_3_)‒	OCH_3_	-	5.62
39	(*S*,*R*)-**8** [3]	A	*tert*C_4_H_9_S^+^*(O^−^)NHC**H(CH_3_)‒ [*(*S*), **(*R*)]	OCH_3_	-	5.61
40	**12l** [2]	A	*cyclo*-C_6_H_11_-CH_2_NHCH(CH_3_)‒	OCH_3_	-	5.61
41	**14b** [2]	B	Ph(CH_2_)_2_NHCH(CH_3_)‒	OCH_3_	H	5.60
42	**14e** [2]	B	CH_3_(CH_2_)_3_NHCH(CH_3_)‒	OCH_3_	H	5.49
43	**12j** [2]	A	CH_3_(CH_2_)_3_NHCH(CH_3_)‒	OCH_3_	-	5.38
44	**12h** [2]	A	Ph(*E*)CH=CHCH_2_NHCH(CH_3_)‒	OCH_3_	-	5.36
45	**12i** [2]	A	CH_3_NHCH(CH_3_)‒	OCH_3_	-	5.09
46	**9a** [2]	A	PhCH_2_OCH(CH_3_)‒	OCH_3_	-	5.02
47	**12m** [2]	A	*N*-morpholino-CH(CH_3_)‒	OCH_3_	-	5.00
48	**11a** [2]	A	PhCONHCH(CH_3_)‒	OCH_3_	-	4.94
49	(*R*,*S*)-**8** [3]	A	*tert*C_4_H_9_S^+^*(O^−^)NHC**H(CH_3_)‒ [*(*R*),**(*S*)]	OCH_3_	-	4.80
50	**12n** [2]	A	*N*-pyrrolidino-CH(CH_3_)‒	OCH_3_	-	4.79
51	**1** [6]	A	ageloyloxy-CH(CH_3_)‒	OCH_3_	-	4.72
52	**11b** [2]	A	PhCH_2_CONHCH(CH_3_)‒	OCH_3_	-	4.51
53	**9b** [2]	A	Ph(CH_2_)_2_OCH(CH_3_)‒	OCH_3_	-	4.22

^a^ Compound **12k** in reference [3] was obtained and tested as racemic mixture. Two of the four possible stereoisomers, differing in the configuration at C-3′ of the side chain, were included as separate structures in the current model, both (*R*)-configured at C-1′, consistent with the other compounds. * Definition of chiral center denoted * in the same line. ** Definition of chiral center denoted ** in the same line.

**Table 2:** Comparison of the statistical quality of the 3D-QSAR models obtained with the non-protonated molecules in alignment B (Figure 1) and 20 different training/test set divisions. For a detailed description of the training/test set divisions, see Table S1.

Model	PC	*R*^2^ (SDEC)	F-Test	*Q*^2^ (SDEP_int_)	*P*^2^ (SDEP_ext_)	Progressive Scrambling (Critical Value: 0.85; Fit Order: 2) [8]	
						Fitted *Q_s_*^2^*	*Q*_0_^2^*
A	5	0.9935 (0.0733)	1006.9488	0.7847 (0.4212)	0.4507 (0.6872)		
B	5	0.9820 (0.1254)	360.5910	0.8099 (0.4080)	0.3893 (0.6749)		
**C**	**5**	**0.9779 (0.1303)**	**291.6092**	**0.7531 (0.4354)**	**0.6760 (0.6094)**	**0.5933**	**0.6980**
D	5	0.9750 (0.1406)	257.3185	0.7241 (0.4670)	0.7385 (0.5461)		
**E**	**5**	**0.9801 (0.1294)**	**325.3210**	**0.7680 (0.4420)**	**0.6713 (0.5280)**	**0.6349**	**0.7469**
F	5	0.9926 (0.0817)	891.0630	0.8849 (0.3233)	0.2737 (0.6719)		
**G**	**5**	**0.9796 (0.1306)**	**316.6972**	**0.8126 (0.3957)**	**0.6935 (0.5140)**	**0.6284**	**0.7393**
H	4	0.9737 (0.1485)	314.1282	0.8029 (0.4062)	0.5954 (0.5892)		
I	4	0.9610 (0.1806)	209.2875	0.6316 (0.5547)	0.7668 (0.4477)		
**J**	**5**	**0.9805 (0.1275)**	**332.5837**	**0.8040 (0.4047)**	**0.6968 (0.5097)**	**0.6307**	**0.7420**
**K**	**5**	**0.9806 (0.1273)**	**333.7178**	**0.8139 (0.3943)**	**0.7076 (0.5018)**	**0.6501**	**0.7648**
L	5	0.9797 (0.1302)	318.8622	0.7008 (0.5001)	0.6731 (0.5304)		
**M**	**5**	**0.9806 (0.1294)**	**334.3370**	**0.7922 (0.4240)**	**0.6600 (0.5099)**	**0.6157**	**0.7244**
N	5	0.9802 (0.1310)	326.0172	0.8126 (0.4026)	0.5481 (0.5882)		
O	3	0.9186 (0.2605)	131.6544	0.7038 (0.4969)	0.5791 (0.6035)		
P	5	0.9791 (0.1346)	309.5729	0.8096 (0.4064)	0.4710 (0.6365)		
Q	5	0.9852 (0.1111)	440.3674	0.7923 (0.4165)	0.5436 (0.6282)		
R	5	0.9909 (0.0836)	721.4217	0.8681 (0.3190)	0.5098 (0.7076)		
S	5	0.9928 (0.0755)	903.8373	0.8857 (0.2999)	0.5039 (0.6936)		
**T**	**5**	**0.9810 (0.1264)**	**341.2076**	**0.7502 (0.4587)**	**0.6616 (0.5324)**	**0.5995**	**0.7053**
mean	4.8	0.9782 (0.1289)	423.3272	0.7853 (0.4188)	0.5905 (0.5855)		

## Data Availability

All data underlying and resulting from this study are available in electronic form from the corresponding author.

## Supplementary Materials

The following are available online: Figure S1: Plot of predicted vs. experimental pIC_50_ values of model K. Table S1: Test sets in models A–T.

## Sample Availability

No samples are available from the authors.
