# Supplementary material for: Quantitative Structure–Activity Relationships of Natural-Product-Inspired, Aminoalkyl-Substituted 1-Benzopyrans as Novel Antiplasmodial Agents
Source: Molecules. 2021 Aug 30;26(17):5249. doi: 10.3390/molecules26175249 (PMC8434475; doi:10.3390/molecules26175249)
Supplement: Supplementary file 1 [file molecules-26-05249-s001.zip › molecules-1312422-supplementary.pdf]

molecules

Supplementary Materials

Quantitative Structure-Activity Relationships of Natural-Product-Inspired, Aminoalkyl-Substituted 1-Benzopyrans as Novel Antiplasmodial Agents

Friederike M. Wunsch[1], Bernhard Wünsch[2] , Freddy A. Bernal[1,3] and Thomas J. Schmidt[1,*]

[1] Institute of Pharmaceutical Biology and Phytochemistry (IPBP), PharmaCampus, Corrensstrasse 48, D-48149 Münster, Germany; Friederike.Wunsch@gmx.de (F.M.W.); thomschml@uni-muenster.de (T.J.S.)
[2] Institute of Pharmaceutical and Medicinal Chemistry (IPMC), PharmaCampus, Corrensstrasse 48, D-48149 Münster, Germany; wuensch@uni-muenster.de
[3] Current Address: Transfer Group Anti-infectives, Leibniz Institute for Natural Product Research and Infection Biology, HKI, Beutenbergstraße 11a, 07745 Jena, Germany; Freddy.Bernal@hki-jena.de
* Correspondence: thomschm@uni-muenster.de; Tel.: +49 251 83 33378

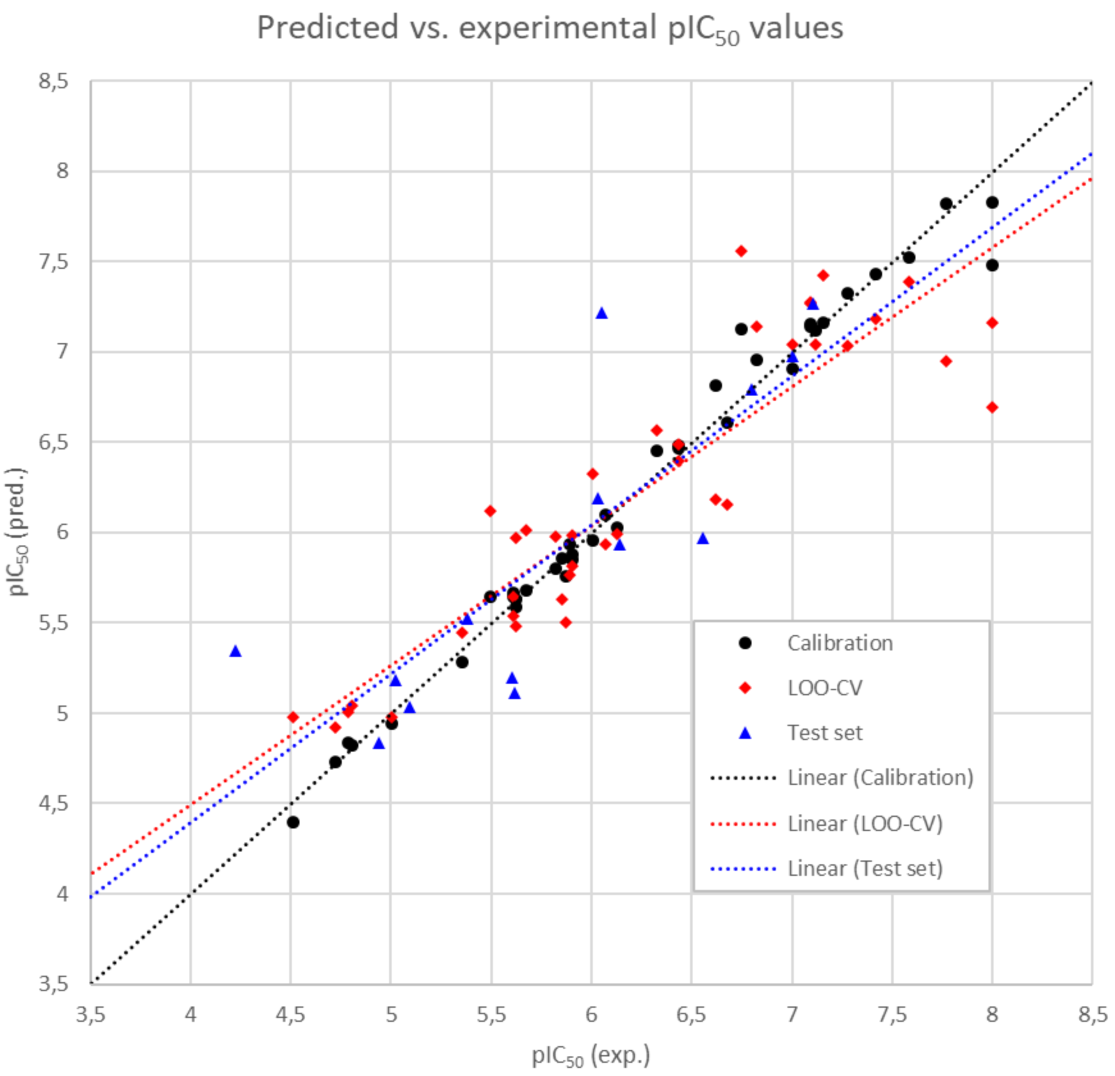

Figure S1. Plot of predicted vs. experimental pIC_{50} values of model K.

Table S1. Test sets in models A-T.

Model	Test set compounds (n=14 ≈ 26%)
A	2, 5, 19, 24, 25, 26, 27, 31, 33, 38, 42, 45, 49, 52
B	10,14, 18,19, 23, 25, 27, 30, 38, 42, 45, 46, 50, 52
C	9, 13, 27, 30, 32, 34, 38, 40, 42, 45, 47, 49, 50, 53
D	19, 23, 27, 30, 32, 34, 38, 40, 42, 45, 47, 49, 50, 53
E	10, 14, 15, 19, 24, 26, 27, 38, 41, 43, 45, 46, 48, 53
F	2, 10, 19, 24, 26, 27, 30, 31, 38, 39, 41, 42, 45, 48
G	9, 13, 15, 19, 24, 26, 27, 38, 41, 43, 45, 46, 48, 53
H	9, 13, 17, 18, 24, 26, 27, 38, 41, 43, 45, 46, 48, 53
I	9, 13, 15, 19, 22, 27, 28, 38, 41, 43, 45, 46, 48, 53
J	9, 13, 15, 19, 22, 26, 27, 38, 41, 43, 45, 46, 48, 53
**K**	9, 13, 15, 19, 23, 26, 27, 38, 41, 43, 45, 46, 48, 53
L	9, 13, 15, 19, 24, 26, 27, 37, 40, 43, 45, 46, 48, 53
M	9, 13, 15, 19, 24, 26, 27, 32, 38, 41, 44, 46, 48, 53
N	9, 13, 15, 19, 24, 26, 28, 32, 38, 41, 44, 46, 48, 53
O	9, 13, 14, 18, 24, 26, 27, 38, 41, 43, 45, 46, 48, 53
P	9, 13, 15, 19, 24, 26, 27, 38, 41, 43, 45, 46, 47, 52
Q	9, 13, 15, 19, 24, 26, 31, 35, 41, 43, 45, 46, 48, 53
R	2, 10, 15, 19, 24, 26, 27, 38, 41, 43, 45, 46, 48, 53
S	2, 10, 15, 19, 23, 26, 27, 31, 38, 41, 45, 46, 48, 53
T	9, 13, 15, 19, 24, 26, 27, 33, 36, 43, 45, 46, 48, 53
